# Improved Expression Systems for Regulated Expression in *Salmonella* Infecting Eukaryotic Cells

**DOI:** 10.1371/journal.pone.0023055

**Published:** 2011-08-01

**Authors:** Carlos Medina, Eva María Camacho, Amando Flores, Beatriz Mesa-Pereira, Eduardo Santero

**Affiliations:** Centro Andaluz de Biología del Desarrollo, CSIC/Universidad Pablo de Olavide, Seville, Spain; Instituto Butantan, Brazil

## Abstract

In this work we describe a series of improvements to the *Salmonella*-based salicylate-inducible cascade expression system comprised of a plasmid-borne expression module, where target gene expression is driven by the P_m_ promoter governed by the XylS2 regulator, and a genome-integrated regulatory module controlled by the *nahR*/P*_sal_* system. We have constructed a set of high and low-copy number plasmids bearing modified versions of the expression module with a more versatile multiple cloning site and different combinations of the following elements: (i) the *nasF* transcriptional attenuator, which reduces basal expression levels, (ii) a strong ribosome binding site, and (iii) the Type III Secretion System (TTSS) signal peptide from the effector protein SspH2 to deliver proteins directly to the eukaryotic cytosol following bacterial infection of animal cells. We show that different expression module versions can be used to direct a broad range of protein production levels. Furthermore, we demonstrate that the efficient reduction of basal expression by the *nasF* attenuator allows the cloning of genes encoding highly cytotoxic proteins such as colicin E3 even in the absence of its immunity protein. Additionally, we show that the *Salmonella* TTSS is able to translocate most of the protein produced by this regulatory cascade to the cytoplasm of infected HeLa cells. Our results indicate that these vectors represent useful tools for the regulated overproduction of heterologous proteins in bacterial culture or in animal cells, for the cloning and expression of genes encoding toxic proteins and for pathogenesis studies.

## Introduction

In recent years, a range of regulated prokaryotic gene expression systems have been developed to adapt production conditions for different requirements. Most systems consist of a single module containing both an inducible transcriptional regulator, responsive to an exogenous factor, and an expression element containing the gene of interest under the control of a promoter that triggers gene expression in response to the regulatory element. Successive modifications to existing protein production systems have been made in order to improve their performance in aspects such as reducing basal expression without compromising maximal expression levels, something especially useful for expressing or cloning genes encoding proteins with deleterious effects. Mutated promoters and regulators, low gene dosage and transcriptional attenuation have been extensively explored in an effort to reduce undesired basal expression levels [1], [2], [3], [4]. Although the highest levels of recombinant protein expression are obtained by amplifying gene dosage with high-copy plasmids, competition between exogenous expression and host cell metabolism can lead to cell growth inhibition [5]. Growth effects or low-level toxicity can lead to selection pressure within the bacterial population for changes, which negatively affect recombinant protein expression. The loss of protein expression competence when the encoding genes are located in multi-copy vectors represents a major problem in situations where plasmid selection is not possible, such as regulated protein expression during bacterial infection.

The efficiency of these single systems may be increased by coupling at least two transcriptional regulators in a cascade, where expression of one regulator directs expression of a second regulator, which in turn directs transcription of the heterologous gene located at the expression module. These transcriptional regulatory cascades substantially amplify the amount of protein produced in natural [6] or artificial systems [7], [8]. Expression systems based on a regulatory cascade have been constructed that take advantage of the inducing activity of salicylate on two transcriptional activators derived from *Pseudomonas putida*: NahR, from the naphalene degradation plasmid NAH7, and XylS2 from the toluene/xylene degradation plasmid pWW0 [7], [9]. The binding of salicylate to NahR and XylS2 activates the expression of genes controlled by the P_sal_ and P_m_ promoters respectively. The regulatory module combines *nahR* with *xylS2* under the control of the NahR-responsive P_sal_ promoter. The gene of interest is inserted into a separate expression module downstream of the XylS2-responsive P_m_ promoter. In the absence of salicylate both NahR and XylS2 are inactive, resulting in very low basal expression of P_m_-controlled target genes due to low levels of *xylS2* expression and the absence of salicylate-dependent XylS2 activation. Conversely, the presence of salicylate strongly activates target gene expression in two ways, by binding NahR and activating P_sal_ *-xylS2* transcription and then by inducing XylS2 to drive P_m_ promoter-mediated expression. Expression systems based on XylS2 and NahR have been shown to have low basal expression levels together with very high inducing ratios and stable levels of expression [7], [8]. By inserting single copies of both regulatory and expression modules into the host strain chromosome, we have shown that it is possible to stably drive protein expression for several days even without selective pressure, unlike high-copy plasmid based systems [7].

To further reduce basal expression levels, an additional regulatory circuit was also incorporated into this system, consisting of two elements from *Klebsiella oxytoca* involved in nitrate assimilation, *nasF,* a transcriptional attenuator [10], [11], [12], [13] and *nasR,* which encodes the corresponding antiterminator protein that prevents *nasF* transcriptional termination in the presence of nitrate or nitrite. Incorporation of *nasF* downstream of the P_m_ promoter reduces basal transcriptional levels from the expression module [3]. By including a P*_sal_* promoter-controlled *nasR* module on an additional plasmid, salicylate-induction activates the P_m_ *-*promoter via activation of NahR and XylS2 and at the same time alleviates *nasF*-mediated transcriptional termination via NahR-driven *nasR* expression. This regulatory circuit has been successfully tested on *Salmonella enterica* serovar Typhimurium to selectively switch on gene expression during bacterial infection [14]. This system also presents the advantage that it can be induced by acetyl salicylic acid (ASA) [14], one of the most widely used and best-characterized analgesic and anti-inflammatory drugs available [15]. *Salmonella* is an enteric bacterial pathogen that causes a variety of food and water-borne diseases, commonly used as a model to study host-pathogens interactions. Here we demonstrate a series of improvements to the original configuration of the regulatory module by coupling the antiterminator gene *nasR* with P*_sal_*/*xylS2* on the regulatory module, as well as cloning a constitutively-expressed GFP encoding gene downstream. Additionally, we have developed a series of low and high-copy plasmids bearing modified expression modules. Each vector has a particular combination of basal expression level and fully induced level by salicylate or acetyl salicylate, and together they cover a wide range of basal and induced levels of expression. These modifications are useful for a variety of purposes such as providing different expression levels and induction ratios under the same induction conditions by choosing the appropriate vector, tracking bacteria during infection studies, or translocating induced proteins to the eukaryotic cytosol.

## Results

### Construction of a *Salmonella* strain bearing a new regulatory module in its chromosome to overproduce heterologous proteins

**Figure 1:**
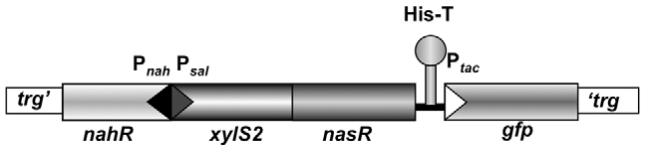
Organization of the improved regulatory module integrated at the *trg* locus of the *Salmonella* chromosome. Boxes represent genes and arrowheads represent promoters. Hairpin represents the histidine operon transcriptional terminator from *Salmonella* (His-T). *nasR* and *xylS2* are coupled in the same transcriptional unit. *nahR* and *xylS2/nasR* are differentially transcribed from P*_nah_* and P*_sal_* respectively. *gfp* is transcribed from P*_tac_*.

The salicylate inducible cascade expression system allows the regulated intracellular expression of proteins in *Salmonella* [14]. We have improved on the regulatory module in two ways: (i) *nasR* was introduced downstream of *xylS2* to coordinate transcription of the activator and antiterminator proteins and avoid the requirement for two plasmids; (ii) a *gfp* gene transcribed from a P*_tac_* promoter was inserted downstream the regulatory module to track *Salmonella* during the infection process, as a tool to study host-pathogen interactions [16], [17], [18]. P*_tac_-gfp* is preceded by a strong transcription terminator [19] to prevent read-through transcription from the regulatory *xylS2* and *nasR* genes, since high levels of GFP in *Salmonella* can reduce their ability to infect eukaryotic cells [20] (Figure 1).

The new regulatory module was inserted into the *trg* locus of *Salmonella enterica* serovar Typhimurium strain 14028 chromosome to create the strain MPO96. This locus was selected because it encodes a ribose/galactose chemoreceptor described as non-essential for bacterial pathogenesis [21].

### Expression vectors design

Since *Salmonella* does not metabolise the inducer, its concentration can be kept constant in the culture medium and it is possible to obtain different expression levels with the same expression vector in *Salmonella* cultures by using different inducer concentrations [9]. However, controlling a particular expression level in an animal model by maintaining constant an intermediate inducer concentration inside the animal is very difficult, above all considering that the inducer is metabolised by the eukaryotic cells. Reproducible control of different expression levels can be obtained by using a saturating inducer concentration and plasmids whose fully induced expression levels were different [22]. To control different expression levels and to obtain a wide range of basal and induced expression levels, we have constructed a set of vectors that produce different levels of protein under the same induction conditions (Table S1).

First, we improved the Multiple Cloning Site (MCS) of the previous multi-copy expression vector pMPO27 [3] with the addition of new restriction sites and the elimination of duplicated ones, thus generating plasmid pMPO57 (MCSII). The whole fragment comprising the *rrnBT1T2* terminators, the P_m_ promoter and the MCSII is flanked by *Not*I restriction sites, allowing for the easy transfer of cloned genes between vectors. A synthetic DNA fragment containing the T7 Shine-Dalgarno (SD) sequence and the upstream A+T rich leader was introduced into pMPO57 to construct plasmid pMPO58. An *Nde*I site downstream of the T7 SD incorporates an ATG start codon can be used to insert coding sequences of interest in-frame. Plasmids pMPO51 and pMPO52, lacking the *nasF* attenuator, were generated from pMPO57 and pMPO58 respectively.

**Figure 2:**
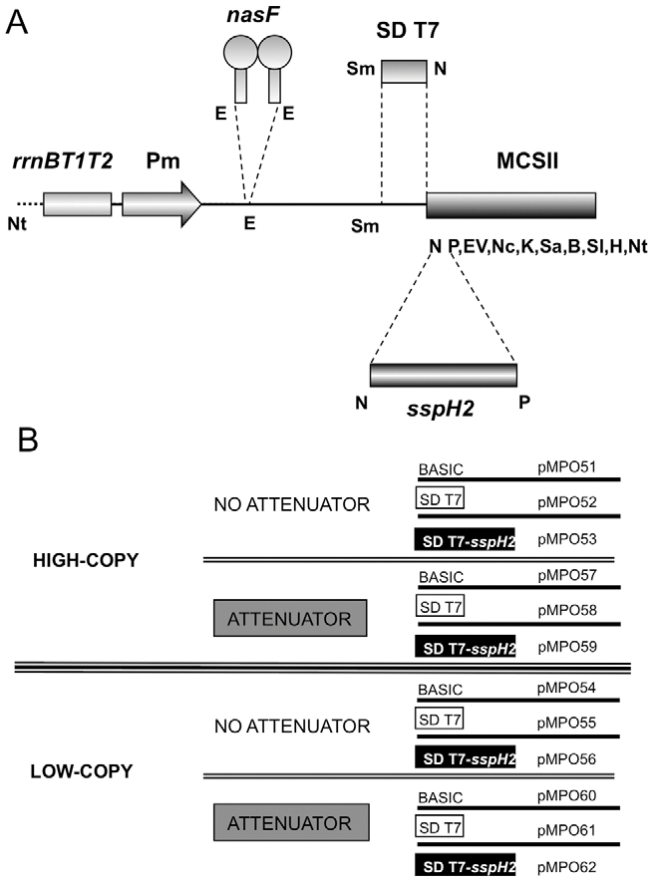
Organization of the different expression modules. (A) Schematic diagram of the different elements of the expression module. *rrnB*T1T2, terminators of ribosomal RNA operons; Pm, P_m_ promoter; *nasF*, transcriptional attenuator; SD T7, Shine-Dalgarno sequence from pT7-7; *sspH2*, type three secretion system signal peptide; MCSII, multiple cloning site. Letters correspond to restriction enzyme sites: Nt, NotI; E, EcoRI; Sm, SmaI; N, NdeI; P, PstI; EV, EcoRV; Nc, NcoI; K, KpnI; Sa, SacI; B, BamHI; Sl, SalI; H, HindIII. (B) Combinations of the main characteristics of the vectors described in this work.

We generated low-copy number versions of these plasmids using the pWSK29 derivative pMPO20 (pMPO54, pMPO55, pMPO60 and pMPO61, respectively) (Figure 2 and Data S1).

Vectors bearing the T7 SD sequence were additionally modified to include the 5′ terminal 450 nucleotides of *sspH2*, thus generating plasmids pMPO53, pMPO56, pMPO59 and pMPO62 (See Figure 2 and Data S1). SspH2 is an effector protein of *Salmonella*, which is secreted to the eukaryotic cytoplasm through the Type III Secretion System (TTSS) of the Salmonella Pathogenicity Island 2 (SPI 2). Fusion with this N-terminal signal peptide is sufficient to direct proteins secretion to the eukaryotic cytoplasm [23], [24].

Finally, we constructed an additional plasmid, pMPO1003 (Data S1), containing the MCSII and a sequence encoding the HA epitope under the control of the P_m_ promoter. This plasmid can be used for the construction of fusion proteins to detect its secretion into eukaryotic cytoplasm using an anti-HA antibody (see below).

### Gene expression rates of the new vectors

In order to test the functionality of the new elements included in the expression and regulatory modules (T7 SD sequence, attenuator, NasR and low gene dosage), we constructed *lacZ* translational fusions in our vectors bearing the T7 SD sequence. To compare expression levels obtained from T7 SD to those obtained with a different SD, we cloned the previously described *cat-lacZ* translational fusion into vectors lacking the T7 SD [25]. Both types of fusions were also transferred to the low-copy number plasmid pMPO20. Thus, we constructed a plasmid collection containing *lacZ* translational fusions with or without *nasF* attenuator, with T7 or *cat* SD sequences and in high or low-copy number (Table 1 and Data S1). The ß-galactosidase assays were performed using the *Salmonella* strain MPO96, which expresses *nasR* from the chromosomally-integrated regulatory module.

**Table 1:** ß-galactosidase expression levels and induction ratios obtained with the different expression vectors in the strain MPO96.

Name	Copy number	Nas F	SD	Expression level (Miller Units)			Induction ratio	
				No induced	+Sal	+Sal+NO_3_	+Sal	+Sal +NO_3_
pMPO94	High	Yes	cat	177±34	13133±1999	14246±898	74	80
pMPO96	High	No	cat	944±55	17593±3807	17380±1180	19	18
pMPO1000	Low	Yes	cat	59±19	4308±290	9156±1402	73	154
pMPO1001	Low	No	cat	297±33	7209±258	7144±377	24	24
pMPO1007	High	Yes	T7	1950±212	76883±9661	75576±4889	39	39
pMPO1005	High	No	T7	8572±482	77608±5719	73615±5729	9	9
pMPO1008	Low	Yes	T7	662±183	49326±1653	71041±4324	75	107
pMPO1006	Low	No	T7	4203±715	70125±4676	72167±4282	17	17

MPO96 bear the *nasR* in the regulatory module. Salicylate induces transcription initiation from the P_m_ promoter, whilst nitrate activates antitermination activity of NasR. Results show the average of three independent experiments.

The results shown in **Table 1** indicate that there was considerable variation in the range of basal or induced levels of protein production between different vectors. The highest level of ß-galactosidase activity following induction (77,608 Miller Units) was obtained using the multi-copy vector lacking the *nasF* attenuator and bearing the T7 SD sequence. In this case the expression level was 18-fold higher than the lowest induced level, obtained with the low-copy configuration bearing the attenuator and lacking the T7 SD. In general, the vectors carrying T7 SD achieved induced activity levels 4 to 10-fold higher than their respective counterparts bearing the *cat* SD.

As expected, the *nasF* attenuator substantially reduced basal expression levels in all vectors. However, upon induction, vectors bearing the attenuator strongly expressed *lacZ,* at levels approximately 70% of their respective counterparts lacking the attenuator. Expression from *nasF* attenuator vectors could be further increased by nitrate addition, resulting in maximal expression levels broadly equivalent to those from vectors lacking the attenuator. These results suggest that NasR produced from the chromosomal regulatory module is effective in preventing transcription termination at the *nasF* attenuator.

The best induction ratios (above 100-fold) were obtained with the low-copy number vectors bearing the *nasF* attenuator. Interestingly, the highest expression level from the low-copy number vector was comparable to that of its corresponding high-copy number vector (See values of ß-gal activitiy obtained with plasmid pMPO1008 versus plasmid pMPO1007 in table 1). This result indicates that using *lacZ* as a reporter gene the use of low-copy vectors increases regulatory capacity without compromising maximal levels of protein production.

**Figure 3:**
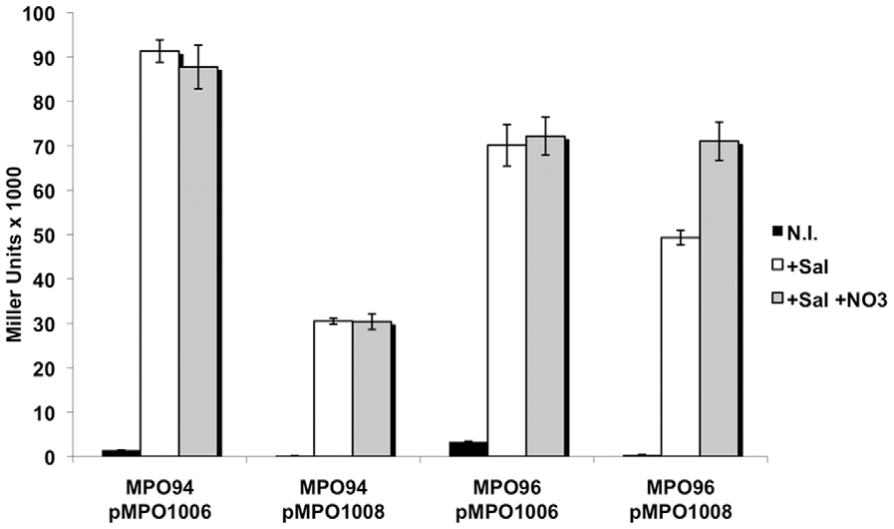
ß-Galactosidase activity in *Salmonella* strains carrying *nasR*. Strain MPO96 with the *nasR* containing chromosomal regulatory module and MPO94 with the non-*nasR* containing version. Strains harbored plasmids with (pMPO1008) or without (pMPO1006) the *nasF* attenuator. Black bars, not induced; white bars, salicylate induced; grey bars, induced by salicylate plus nitrate. Error bars correspond to Standard Deviation. The results show the average of three independent experiments.

To confirm the effectiveness of chromosomally-produced NasR in counteracting *nasF* transcription attenuation, we assayed *lacZ* expression in absence or presence of the attenuator in MPO96 and its isogenic NasR^−^ strain MPO94 (Figure 3) using the low copy plasmids bearing the T7 SD sequence (pMPO1006 and pMPO1008). In the absence of *nasR*, salicylate-induced expression from the *nasF* attenuator vector was 33.4% of that from the vector lacking it and this expression did not change in the presence of nitrate. However, in the NasR^+^ strain, the addition of salicylate increased *lacZ* expression, and was further enhanced by the presence of nitrate up to the levels obtained with the vector lacking *nasF* in the same strain. Thus, the increased expression following nitrate addition is dependent on NasR. In addition, our results show that a single chromosomal copy of NasR under P*_sal_* control is sufficient to block *nasF* attenuator transcriptional termination.

In summary, these results show that using the same induction conditions, this regulatory cascade can produce different amounts of the same protein, depending on the particular features of the expression vector.

### Cloning the *colE3* gene in the expression vectors

Obtaining the lowest possible levels of basal expression is critical when the protein of interest is toxic for the host bacteria. Significant basal expression of a protein that adversely affects bacteria growth or survival is likely to drive selection within the bacterial population for mutant bacteria that do not express a functional protein.

To determine if our vectors have sufficiently low levels of basal expression to allow the cloning of highly cytotoxic genes, we attempted to clone *colE3* (Data S1). This gene codes for colicin E3, a ribonuclease that is cytotoxic to a number of enterobacteria. Strains that naturally produce colicin E3 are protected by the co-expression of the immunity protein ImmE3 [26].

We tried to clone a DNA fragment carrying the *colE3* gene under the control of P_m_ in multi-copy vectors, with or without the *nasF* attenuator, in an ImmE3^+^ *E. coli* strain [27]. In spite of the repeated attempts, no colonies whatsoever were obtained with the vector lacking the attenuator, suggesting that basal expression levels obtained from high-copy number plasmids in the absence of the attenuator are sufficiently high to be toxic even in the presence of its immunity protein. In contrast, numerous colonies were obtained when the ImmE3^+^ *E. coli* strain was transformed with a *nasF*-regulated multi-copy vector (pMPO1009).

Interestingly, similar numbers of clones were obtained when ImmE3^+^ *E. coli* were transformed with low-copy *colE3* plasmids with or without the *nasF* attenuator (pMPO1011 and pMPO1010 respectively). This result indicates that, in the presence of the immunity protein, low gene dosage reduces the basal expression of colicin to a level that allows *colE3* cloning irrespective of the attenuator.

### Maintenance of the colicin production ability in the absence of ImmE3

**Figure 4:**
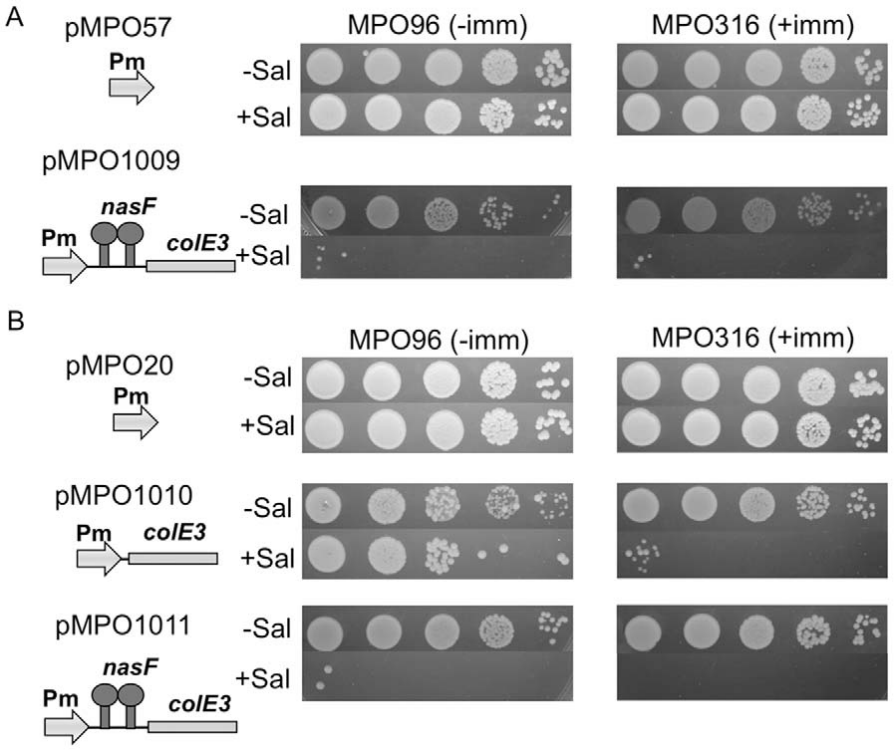
Maintenance and expression of colicine from different plasmid configurations in *Salmonella*. Serial dilutions (10^3^ to 10^7^) of cultures of strains MPO96 and MPO316 expressing *colE3* from high-copy number (A) and low-copy number (B) vectors were plated in LB with or without salicylate and incubated for 24 hs.

Next we decided to test the ability of the *nasF* attenuator to reduce basal expression sufficiently to allow the induced expression of toxic proteins in ImmE3^−^ *Salmonella*. To this end we assayed the capability of the new vectors to produce colicin following salicylate induction both in ImmE3^+^ (MPO316) and ImmE3^−^ (MPO96) *Salmonella* strains. Transformed *Salmonella* were cultured with antibiotic selection and spotted on plates in the presence or absence of salicylate (Figure 4). Strains transformed with *colE3* in multicopy plasmids bearing the *nasF* attenuator showed impaired growth even in the absence of salicylate, as evidenced by the smaller size of their colonies (Figure 4A). Nevertheless, their viability was not compromised since the number of colonies was similar to the control strain transformed with the empty vector. Salicylate induction resulted in the death of the majority of the bacterial population irrespective of the immunity, suggesting that a strong colicin producing capacity had been retained. Only a few colonies tolerated salicylate induction (with a frequency lower than 10^−5^), which presumably represent bacteria (e.g. spontaneous mutants) that produce lower amount of colicin E3 despite antibiotic selection. This observation indicates that the presence of the attenuator reduces basal colicine production sufficiently to maintain cell viability, even in the case of multi-copy plasmids.

Bacteria transformed with the low-copy number plasmid carrying the *nasF* attenuator (pMPO1011) generated healthy fast-growing colonies even in the absence of the immunity *immE3* gene (Figure 4B). Moreover, induction with salicylate killed most of the bacterial population, indicating that the basal expression level had been low enough to allow plasmid-containing bacteria to grow without selecting against colicin production capacity, even in an ImmE3^−^ strain, yet upon induction was able to reach an expression level sufficient to overcome the immunity.

In the absence of the *nasF* attenuator (pMPO1010) most ImmE3+ bacteria retained colicin production capacity upon induction; albeit with a low frequency (≤10^−5^) of bacterial survival, presumably corresponding to bacteria with impaired colicin production. However, growth of the ImmE3^−^ strain was clearly affected even in the absence of salicylate. Interestingly, while most colonies were very small, some large colonies were also evident. Growth in the presence of salicylate indicated that a significant proportion of the population (10^−2^–10^−3^) had been deficient for colicin production. Presumably, the large colonies in the medium lacking salicylate reflect the appearance of bacteria lacking the ability to produce the toxin.

Taken together our data indicate that, the combination of the *nasF* attenuator and low copy plasmids permits bacterial growth while retaining the ability to produce toxic proteins. Interestingly, our findings suggest that the increased basal expression in the absence of *nasF* corresponds to increased selection against production-competent bacteria.

### Maintenance of colicin production capacity in the absence of selective pressure

In situations where antibiotic selection cannot be used to maintain the plasmids, such as in cell culture or animal models, the presence of a toxic protein is likely to quickly drive selection for plasmid loss or otherwise lose the ability to produce the toxin. To test whether the reduced basal expression of our expression vectors was sufficient to overcome this type of negative selection, *Salmonella* bearing salicylate-inducible *colE3* plasmids were grown for 20 generations without antibiotic selection, followed by testing for plasmid maintenance and colicin production.

**Figure 5:**
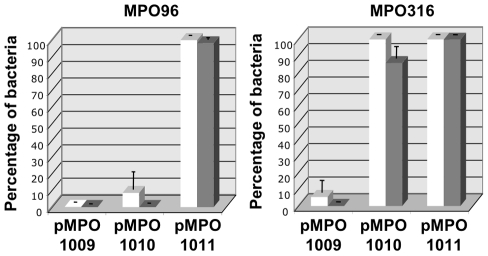
Plasmid stability and expression of functional colicine without antibiotic selection. Percentage of bacteria that retain plasmids (white bars, ampicillin resistance), and salicylate-induced death by accumulation of colicin E3 (grey bars) after 20 generations of growth in non-selective medium. MPO96 - *Salmonella* strain lacking *immE3* gene. MPO316 - isogenic ImmE3^+^ strain. pMPO1009 - high-copy plasmid bearing the attenuator; pMPO1010 and pMPO1011 - low-copy number plasmids that lack or bear the attenuator, respectively. Error bars represent the average of four independent experiments.

As shown in Figure 5, at least 90% of the bacterial population harboring the high-copy vector with the attenuator (pMPO1009) had lost the plasmid and their ability to produce colicin, even when transformed into the immunity-bearing strain. In contrast, the low-copy plasmid lacking the attenuator (pMPO1010) was stably maintained by the ImmE3+ strain and kept its response to salicylate induction intact. However, most ImmE3^−^ bacteria had lost this plasmid (>95%) and did not die upon salicylate induction (100% of the population). Remarkably, when the low-copy colicin-producing plasmid incorporated the *nasF* attenuator (pMPO1011), the majority of the bacterial population maintained the plasmid and died upon salicylate induction (95 to 99%), even in the absence of the immunity protein. These data show that changes in basal expression can dramatically affect the retention of expression-module containing plasmids. Moreover, our results suggest that the combination of a low-copy vector and the presence of the *nasF* is able to reduce basal expression below the selection threshold in highly sensitive ImmE3- *Salmonella*, even in the absence of positive antibiotic plasmid selection.

### Expression in *gfp*-tagged *Salmonella* while infecting eukaryotic cells

**Figure 6:**
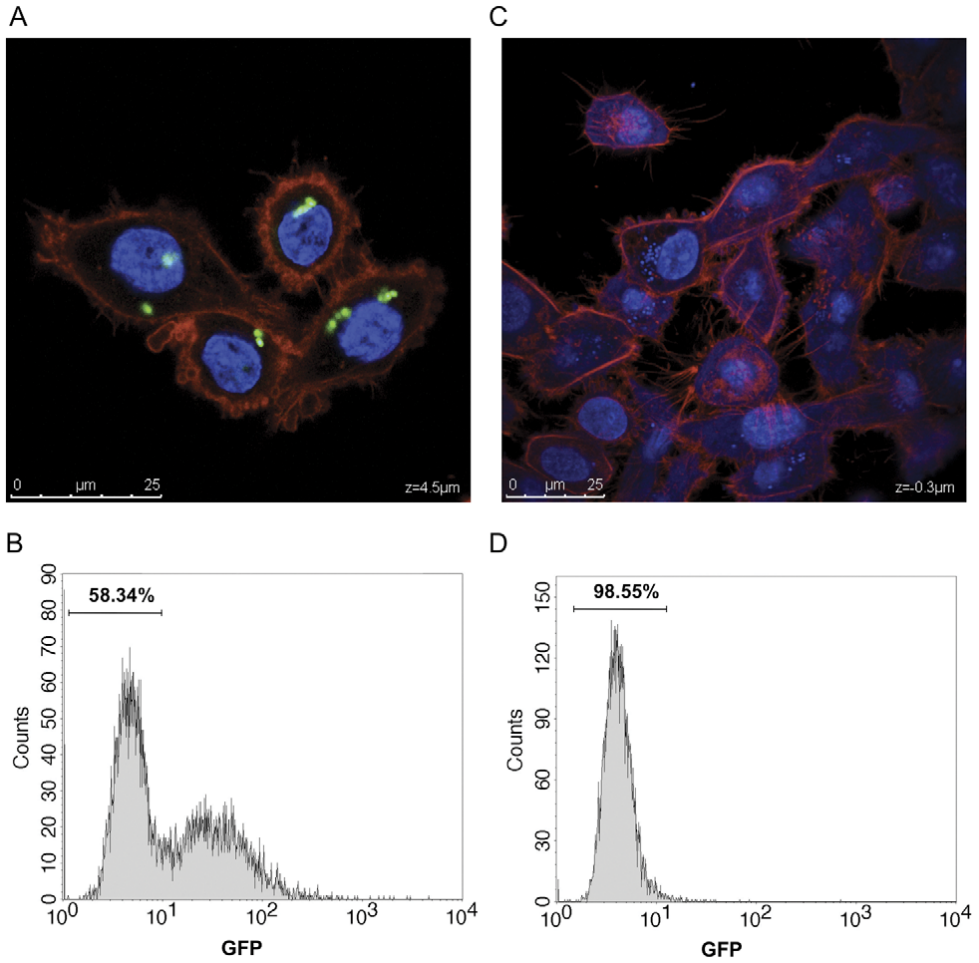
*Salmonella* detection by microscopy and flow cytometry analysis. Visualization of HeLa cells infected with the MPO96 (A) or 14028 (C) strains using the Confocal Microscope Leica SPE (630X). MPO96 strain express GFP (green) while 14028 strain does not. Eukaryotic nuclei and bacterial DNA (blue) were stained with Hoescht and eukaryotic cytoskeleton (red) with rhodamine-phalloidine. All the channels were taken at the same Z plane (Z = 4.5 µm in A or 0.3 µm in C). Scale bar is indicated in the left. Fluorescence of HeLa cells infected with the MPO96 (B) or 14028 (D) strains by flow cytometry analysis. The right peak on B represents the infected cells while the left peak represents the non infected cells. The number above the left peak represents the percentage of non-infected cells. Samples were collected 1 h.p.i. for microscopy and flow cytometry analysis. Results show the average of three independent experiments.

To monitor infection and to detect *Salmonella* inside the eukaryotic cells, the *gfp* encoding sequence under the control of the strong P*_tac_* promoter was inserted downstream of the regulatory module in the same *trg* locus of *Salmonella* (see Figure 1). As shown in Figure 6, the strain MPO96 produces enough GFP for detection by fluorescence microscopy or flow cytometry (Figure 6 A,B), while a control strain lacking GFP does not (Figure 6 C,D).

**Figure 7:**
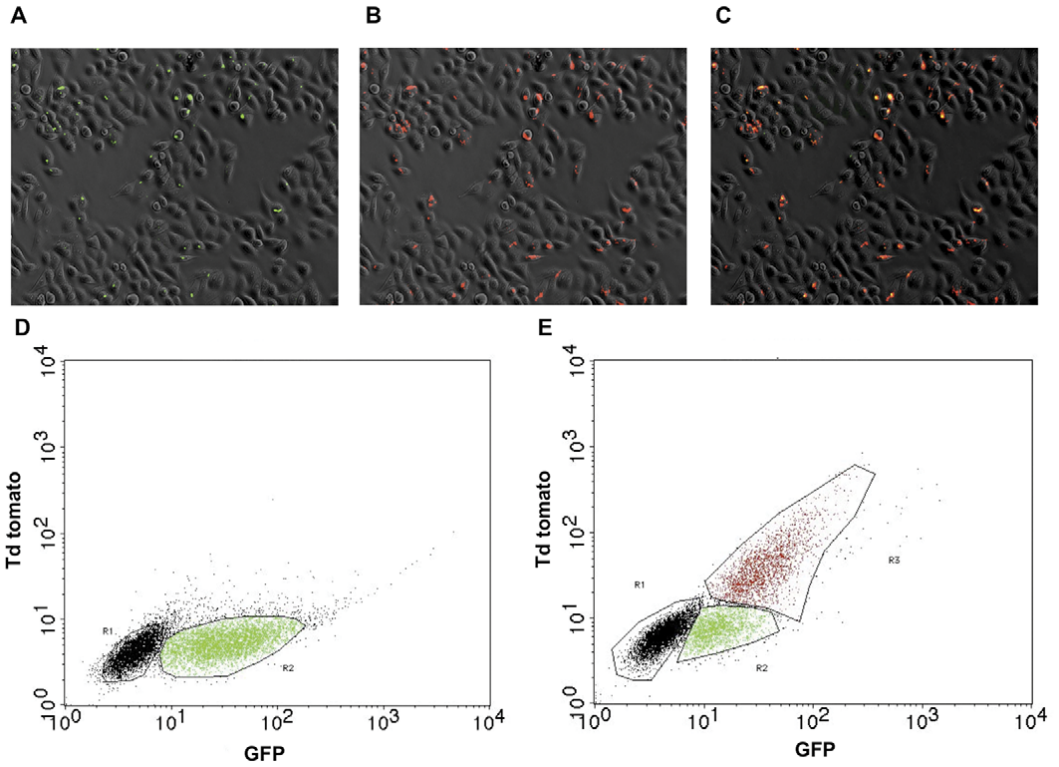
Inducible intracellular expression of dTomato protein. HeLa cells cultures were infected with *Salmonella* MPO94 harbouring pMPO1046 plasmid (100X), induced for 4 hs with 2 mM salicylate and processed for fluorescence microscopy or flow cytometry analysis. Visualization of GFP fluorescence (A), dTomato protein (B), and merge (C) is shown. (D), cytometric analysis of infected and (E) infected and induced cells. R1, not infected cells; R2, Infected cells; R3, Infected and induced cells. Results show the average of three independent experiments.

To quantify the global induction efficiency of the cascade expression system in cell culture, we infected HeLa cells with *Salmonella* strain MPO94 bearing a plasmid (pMPO1046) carrying the dTomato red fluorescent protein encoding gene under P_m_ promoter control. Infected HeLa cultures were induced with salicylate and analyzed by fluorescence microscopy. As shown in Figure 7A–C, the production of the red dTomato protein was limited to those cells infected by *Salmonella*. Quantification by flow cytometry confirmed this result, with 70–80% of GFP positive bacteria expressing the red fluorescent protein upon salicylate induction (Figure 7E), while in the absence of salicylate such expression could not be observed (Figure 7D).

**Figure 8:**
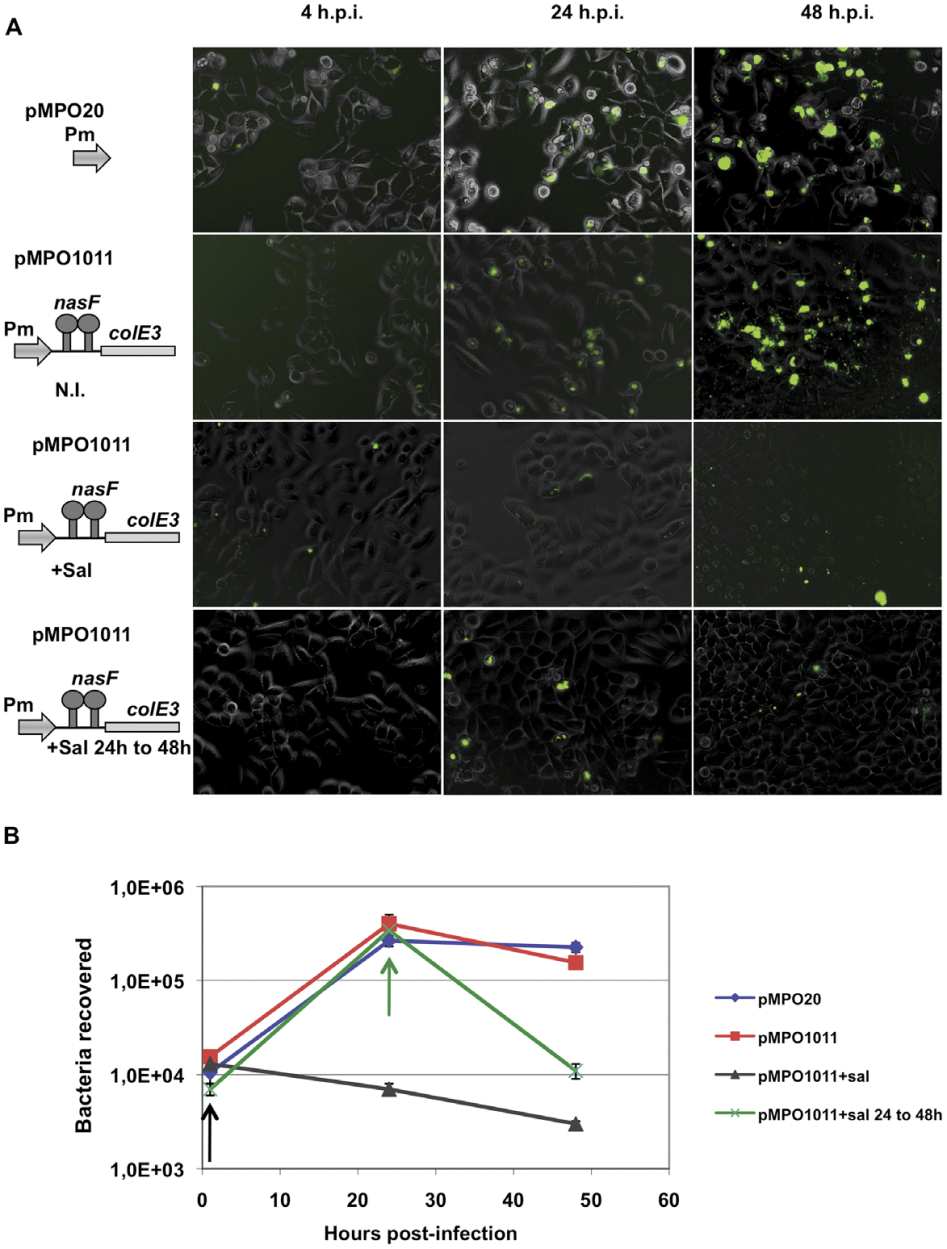
Expression of colicine inside eukaryotic cells. (A) Visualization of HeLa cells (320X) infected by Salmonella MPO96 strain bearing plasmids pMPO20 and pMPO1011 under inducing or no inducing conditions. (B) Bacteria recovered from infected cells at different times post-infection. The absence of GFP inside cells corresponds to bacterial death as demonstrate the decrease of bacteria recovered after salicylate induction. N.I., not induced; +Sal, addition of salicylate at 1 h.p.i.; +Sal 24 to 48 h, addition of salicylate at 24 h.p.i; h.p.i., hours post-infection. Arrows indicate the time of salicylate addition, at 1 h (black) and 24 h (green) post-infection. The data showed in panel A correspond to one representative experiment out of four.

To test the maintenance of cytotoxic protein production capacity when used to infect animal cells, HeLa cultures were infected with an ImmE3^−^ NasR^+^ *Salmonella* (strain MPO96) bearing a low-copy P_m_ *-nasF* attenuator*-colE3* plasmid (pMPO1011). One hour or 24 hours after infection, colicin production was induced by addition of salicylate. At different time points after the infection (1, 24 and 48 hours), infected cells were detached and lysed to count the number of intracellular bacteria. As shown in Figure 8A (upper panels), the combination of the attenuator and low gene dosage permitted bacterial infection and growth in the absence of salicylate. In contrast, the expression of colicin after salicylate induction resulted in bacterial death (lower panels). As shown in Figure 8B, the number of recovered intracellular bacteria remained constant in absence of salicylate. Additionally, the number of bacteria obtained was similar when lysed cultures were plated on LB and LB ampicillin, indicating that the bacteria maintained the plasmid after several hours of growth inside the eukaryotic cell. However, upon salicylate induction the number of c.f.u. decrease significantly showing that bacteria maintain the colicin production capacity at least 24 h after infection. These results are in agreement with those obtained in isolated bacterial cultures, and confirm that the regulated production of toxic proteins can be maintained during the infection process in the absence of selective pressure and antitoxin proteins.

### Regulated *Salmonella* protein secretion into the eukaryotic cell cytoplasm

**Figure 9:**
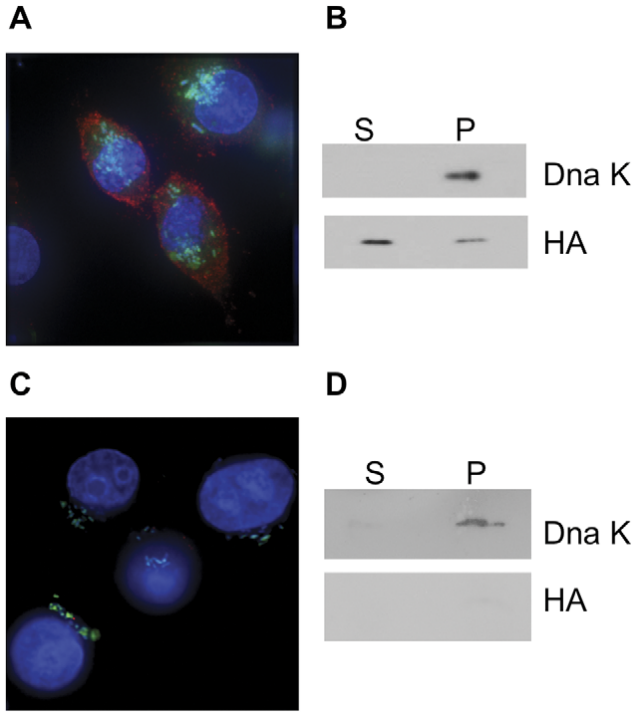
Regulated protein secretion in *Salmonella* through the type three secretion system (TTSS). Secretion of the HA epitope-containing chimeric protein through TTSS (1000X) in the presence (A) or absence of salicylate (C). *Salmonella* strain MPO94 bearing the pMPO1004 plasmid, constitutively expressing GFP (green), HeLa cell nuclei were stained with Hoescht (blue) and anti-HA epitope antibody staining (red). (B) Western blot of supernatant (S) and pellet (P) fractions of HeLa cell cultures infected with the *Salmonella* MPO94 strain bearing the pMPO1004 plasmid in the presence of salicylate or (D) its absence. The HA epitope and the bacterial chaperone DnaK were detected with their corresponding antibodies. The data showed correspond to one representative experiment out of four.

To test if the SspH2 leader peptide cloned in some vectors is able to direct the secretion of fusion proteins from *Salmonella* to the eukaryotic cytosol, the sequence coding for the HA epitope was cloned in the high-copy number, P_m_-T7 SD-*sspH2* plasmid (pMPO53) to generate plasmid pMPO1004. Production of SspH2-HA and its subcellular location were tested in HeLa cell cultures infected with MPO94 containing the pMPO1004 plasmid. As shown in Figure 9A, SspH2-HA was produced by intracellular bacteria after salicylate induction, and secreted into the eukaryotic cell cytoplasm, while in the absence of salicylate the secretion could not be detected (Figure 9C). Quantification of the SspH2-HA fusion protein revealed that 75% of the product was translocated to the eukaryotic cytoplasm only in the presence of salicylate (Figure 9B and D). In contrast, a control strain lacking the *sspH2* sequence in the plasmid was unable to translocate HA (data not shown).

## Discussion

In this work we present modifications that improve the performance of the previously described P_m_-*nasF* NahR-XylS regulatory cascade expression system at two levels: (i) the regulatory module has been modified to include *nasR* under P*_sal_* control and a constitutively expressed *gfp* to allow bacterial infection to be followed; (ii) the expression module has been placed in high and low-copy number plasmids, which were modified to obtain more versatile expression vectors.

The cascade regulatory circuit has been successfully used to express proteins in *E. coli* [3], [7], [8], but since *Salmonella enterica* offers additional possibilities such as regulated expression inside animal cells [14], we have modified the system to adapt it for alternative uses in these bacteria, particularly for its use within animal cells. For this purpose we integrated the new regulatory module into the *Salmonella* chromosome at the *trg* locus, described as non-essential for invasiveness or proliferation [21]. In this module *nasR* and *xylS2* are simultaneously induced by salicylate, avoiding the use of two different plasmids [3]. Although the copy number of the antiterminator *nasR* is reduced in this chromosomal location as compared to the plasmid location, it appears that a single copy of *nasR* is sufficient to achieve efficient antitermination of *nasF*. (Table 1 and Figure 3).

GFP production from a single copy P*_tac_* promoter did not affect performance of the strain (data not shown) but was sufficient to track bacteria during infection processes by microscopy or flow cytometry analysis (Figure 6). This tool offers the possibility of quantifying the fluorescence at different stages of infection, serving as a measure of bacterial proliferation inside eukaryotic cells.

Modifications to the expression module in both low- and high-copy vectors include a more versatile multicloning site, a strong SD sequence to drive efficient translation, a transcriptional attenuator from the *nasF* operon to reduce basal expression levels, and the *sspH2* signal peptide sequence to secrete proteins through the TTSS. With these vectors, we could obtain high induction ratios (Table 1). Moreover, it was possible to obtain different expression levels under the same fully inducing conditions by choosing vectors with different copy numbers or SD sequences with different translation efficiencies [28].

For applications involving the expression of proteins with deleterious effects on the producing bacteria, it is essential that basal expression be tightly-controlled under non-induced conditions to permit the stable maintenance of their expression ability. The low-copy number vectors containing the *nasF* attenuator reduce basal expression level so efficiently that they allowed the maintenance and regulated expression of the cytotoxic *colE3* gene in a *Salmonella* strain even in the absence of the immunity protein that prevents its function (Figure 4). In spite of this, the maximal level of induced expression was not compromised (Table 1). The stable maintenance of colicin-expressing low copy plasmids in the absence of their cognate immunity protein system has been described previously using a system based on the lactose promoter/operator [2]. However, this system presents the disadvantage that as it uses a negative control system, meaning that there is transient target gene expression upon transformation until the repressor, encoded in the same plasmid, has time to be produced in sufficient quantities. In contrast, the regulatory cascade described here uses a positive control system, whereby expression is dependent on the presence of salicylate.

Using the 5′ region of *sspH2*, we have developed expression vectors able to translocate fusion proteins into the eukaryotic cytoplasm (Figure 9). Such translocation depends on the secretion system located in SPI-2 that is only activated under intracellular conditions [29]. Although it has been hypothesized that the overexpression of chimeric SspH2 could saturate the TTSS translocation machinery [30], our result clearly indicates that the translocation capacity of the system is sufficiently high to secrete around 75% of the product generated by this expression system in infected cells.

In addition, we have developed a plasmid (pMPO1003) that can be used to study protein secretion based on the immunodetection of the commonly used HA epitope. This plasmid is conceived for the construction of chimeras with putative effector proteins in order to evaluate their translocation and co-localization. Different systems based on secretion and quantification of β-lactamase cleavage of a specific fluorescent substrate have been previously described to detect protein translocation [31], but its use is technically more difficult due to the instability of the product.

It has been previously reported that in animals *Salmonella* preferentially accumulates in the tumours of affected animals [32], [33]. The combination of controlled production of cytotoxic proteins with the ability to direct its translocation into the eukaryotic cytosol using a TTSS, suggests that this system has the potential to be excellent tool for the delivery of therapeutic drugs into tumors. The regulatory module has been inserted in the *trg* locus, whose mutation does not affect pathogenesis. Interestingly, it has been recently reported that a Trg^−^ *Salmonella* accumulated in the tumour quiescence area and had a higher apoptotic effect on infected cells than a wild type strain [34].

In summary, we present here a series of improvements to the salicylate-based NahR-XylS2 cascade expression system that show great potential for applications in different research areas, such as cloning toxic proteins from different origins, studies of *Salmonella* pathogenesis by expressing genes at different stages during infection, assaying the translocation of putative effector proteins, and specifically targeting the delivery of cytotoxic drugs into tumours.

## Materials and Methods

### Strain, plasmids and growth conditions

All plasmids and bacterial strains used in this work are described in the table S1. Cultures were grown aerobically at 180 r.p.m. and 37°C in LB medium and supplemented when necessary with ampicillin (100 µg/ml), kanamycin (20 µg/ml) or chloramphenicol (15 µg/ml).

### Molecular biology general procedures

All DNA manipulations were performed following standard protocols [35]. The *sspH2* signal peptide and the dTomato genes were PCR-amplified using genomic DNA from *Salmonella* or pRSETB dTomato plasmid as templates, respectively, and primers sspH2–1 (5′ gatatacatatgccctttcatattggaag 3′) and sspH2–3 (5′ tatactgcagcccggatgccccttc 3′) or Tomato-NdeI F (5′ tacatatggtgagcaagggcgagg 3′) and Tomato-HindIII R (5′ taaagcttttacttgtacagctcgtcc 3′) respectively. PCR was performed with High-Fidelity polymerase (Roche) as directed by the manufacturer. The MCSII, T7 SD sequence and the 2xHA-epitope, were constructed by cloning two annealed complementary oligonucleotides (73, 39, and 63 nucleotides long, respectively) into appropriate vectors. The HA (human influenza hemaggulitin) epitope tag is well-characterized and highly immunoreactive whose recognized sequence is YPYDVPDYA. The oligonucleotides sequences are detailed below.

MCS+ (5′ gttcacccgggcatatggcaagaactgcagatatccatggtaccgagctcggatccgtcgacaagcttacttg 3′) MCS- (5′ caagtaagcttgtcgacggatccgagctcggtaccatggatatctgcagttcttgccatatgcccgggtgaac 3′)

SD pT7-7+ (5′ gggaaataattttgtttaactttaagaaggagatataca 3′)

SD pT7-7− (5′ tatgtatatctccttcttaaagttaaacaaaattatttccc 3′)

Sal-HA-Hind+ (5′ tcgacgtacccatacgatgttcctgactatgcgtacccatacgatgttcctgactatgcgtaa 3′)

Sal-HA-Hind− (5′ agctttacgcatagtcaggaacatcgtatgggtacgcatagtcaggaacatcgtatgggtacg3′)

### Determination of ß-galactosidase activity

The plasmids pMPO94, pMPO96, pMPO1005, pMPO1007 (high-copy number) and pMPO1000, pMPO1001, pMPO1006 and pMPO1008 (low-copy number), were used to transform *Salmonella* strains MPO94 and MPO96. After growing overnight in LB with ampicillin as necessary, cultures were diluted 50-fold in the same media, and allowed to grow to OD_600_ 0.2–0.3 before inducing with 2 mM salicylate and in some cases 0.2 g/l sodium nitrate. 2 mM salicylate is within the therapeutic concentration in blood [22] and is sufficient to fully induce the expression system [14]. All cultures were incubated at 37°C and 180 r.p.m. for 3 h after which ß-galactosidase activity was measured as described [36].

### Survival of bacterial populations upon overproduction of colicin E3

To determine the effect of producing colicin E3, *Salmonella* strains MPO96 and MPO316, were transformed by electroporation with the plasmids pMPO57, pMPO1009, pMPO20, pMPO1010 and pMPO1011, grown overnight and diluted 50-fold until OD_600_ 0.5–0.6 was reached. Bacterial survival frequency was estimated by plating spots of different dilutions of the cultures on LB plates alone or with 2 mM salicylate, and estimating growth after incubation for 24 h at 37°C. To estimate the maintenance of colicin production capacity in the absence of selective pressure serial batch cultures were performed as follows. Bacteria were inoculated overnight in the presence of ampicillin; cultures were then diluted 100 fold every 12 hours three times in fresh LB medium. Finally, samples were collected at 0.8 OD_600_ (20 generations estimated), diluted and plated in LB, LB with ampicillin and LB contaning 2 mM salicylate plates. The proportion of plasmid containing bacteria was calculated as c.f.u. obtained in LB with ampicillin respective to LB. The population of bacteria expressing *colE3* was calculated as c.f.u. obtained in LB plates minus c.f.u. obtained in LB salicylate plates.

### Regulatory module integration into the *Salmonella* chromosome

To integrate DNA sequences into the *Salmonella* chromosome we used a modification of the method published by Datsenko and Wanner [37] to inactivate chromosomal genes in *E. coli*, as described in [14]. Kanamycin or chloramphenicol resistance genes were cloned into plasmids bearing the sequences to be integrated at the chromosome (pMPO64, pMPO83 and pMPO1035). Since this new regulatory module is longer than 7 kb, the integration was made sequentially. As a first step, *nahR/XylS2-Cm* was integrated into the *trg* locus of the *Salmonella* chromosome. Later, in two sequential steps, *nasR* and P*_tac_-gfp* were added at the regulatory module downstream from *xylS2*. Finally, the Cm resistance gene was changed to Km, generating the MPO96 strain. Alternatively, *nasR* was deleted to obtain the MPO94 strain.

### *In vitro* bacterial infection of HeLa cells

HeLa cells were grown at 37°C and 5% CO_2_ in tissue culture plates (Nunc) in Dulbecco's modified Eagle's medium (DMEM; Sigma) supplemented with 2 mM L-glutamine and 10% Foetal Calf Serum (FCS) containing a Penicillin-Streptomycin mixture.

Infection experiments were performed essentially as described [38] with minimal modifications. Cells were cultured in 24-well plates at a density of 2×10^5^ cells per well 20 hours before infection. An overnight *Salmonella* culture was diluted 1∶33 on fresh LB and incubated at 37°C during 3.5 h. Cells were equilibrated for 15 min in Earle's buffered salt solution (EBSS) before infection. Bacteria were added at a m.o.i. of 50∶1 allowing the infection to proceed for 15 min at 37°C and 5% CO_2_. Wells were washed twice with Phosphate Buffer Saline (PBS) and incubated for 1 hour with DMEM containing 100 µg/ml gentamicin to kill extracellular bacteria. After that, the antibiotic concentration was reduced to 16 µg/ml. Bacterial induction was performed as described for bacterial cultures, adding 2 mM salicylate to the culture medium and incubating for an additional 4 h. For enumeration of intracellular bacteria, cells were PBS washed, lysed with 0.1%Triton X-100 for 10 minutes and plated on to LB or LB with ampicillin.

An inverted fluorescence microscope (Leica Systems) was used to directly visualize infected cells. For quantitative analysis, cells were detached with trypsin and analyzed by flow cytometry using a FACScalibur cytometer and CellquestPro software (Becton-Dickinson). When necessary, cells were fixed in 4% Paraformaldehyde (PFA) for 20 minutes at room temperature. Cells were then rinsed five times in PBS and incubated with Hoescht and rhodamine-phalloidine for one hour at room temperature. Samples were mounted on glass coverslips and visualized with a confocal microscope Leica SPE.

### Microscopy Assay for translocation of proteins into live eukaryotic cells

Cells previously infected with MPO94 bearing pMPO1004 were induced as described before. After 4 h cells were washed and fixed with PFA 4% during 30 min, permeabilized with 0.1% triton X-100, and incubated for 45 min with blocking buffer (3% FCS in PBS) to inactivate HeLa membrane receptors. Cells were then incubated with 1∶500 anti-HA primary antibody (COVANCE) in blocking buffer overnight. Cells were then washed and incubated for 90 min with a 1∶300 dilution in PBS of anti-Mouse IgG conjugated to Alexa555 secondary antibody (Molecular Probes/Invitrogen). To stain cellular nuclei, Hoescht was added 20 min before washing. Preparations were thoroughly washed with PBS before visualizing by microscopy. Translocation was considered positive when the fluorochrome-conjugated secondary antibody (red) was clearly visible in the cellular cytoplasm outside the bacteria (green).

### Western blot analysis to test bacterial protein secretion into live eukaryotic cells

Cells previously infected with MPO94 bearing pMPO1004 were induced as described before. After 4 h cells were detached by trypsin treatment, washed twice with PBS and then resuspended on 100 µl lysis buffer [39] and kept on ice for 30 minutes. The cell lysate was centrifuged at 20,000 g for 10 minutes, the supernatant was retired and stored, whereas the pellet was washed with 1 ml of PBS and finally resuspended in an original volume of lysis buffer (100 µl). Protein concentrations were determined by the BCA protocol (Sigma) and 20 µg of protein from the pellet and an equivalent volume of supernatant were loaded for SDS-PAGE. After electrophoresis and Western blotting, transference of immunoreactive products to anti-HA were detected by the enhanced chemiluminescence system (Thermo Scientific) and quantified on a Typhoon 9410 scanner using the ImageQuant software (Amersham). DnaK was used as a control to confirm the absence of bacteria in the supernatant.

## Supporting Information

Table S1 **Strains and plasmids used in this work.**
(DOC)

Data S1 **Construction of expression vectors,** ***lacZ*** **fusions and** ***colE3*** **cloning.**
(DOC)
