# Supplementary material for: Improved Expression Systems for Regulated Expression in Salmonella Infecting Eukaryotic Cells
Source: PLoS One. 2011 Aug 1;6(8):e23055. doi: 10.1371/journal.pone.0023055 (PMC3148252; doi:10.1371/journal.pone.0023055)
Supplement: Table S1 — Strains and plasmids used in this work. (DOC) [file pone.0023055.s001.doc]

**Supplementary** **Table.** Strain and plasmids

|  | Characteristics | Reference |
| --- | --- | --- |
| ***E. coli* strains** |  |  |
| *DH5α* | *deoR endA1 gyrA96 recA1 supE44* | [1] |
| HB101 *immE3* | HB101 with a Km mini-Tn5 bearing *immE3* | [2] |
| ***S. typhimurium* strains** |  |  |
| 14028 | Wild type strain | [3] |
| MPO94 | 14028 ∆*trg:: nahR/*P*_sal_–xylS2* /P*_Tac_–gfp* fusion | This work |
| MPO96 | 14028 ∆*trg:: nahR/*P*_sal_–xylS2–nasR*/P*_Tac_–gfp* fusion | This work |
| MPO316 | MPO96 ∆*sifA*::*immE3* | This work |
| **Plasmids** |  |  |
| pRSETB d-Tomato | Source of 852-bp NdeI-HindIII d-Tomato DNA | [4] |
| pCP20 | ApR, CmR, Ts (30ºC) | [5] |
| pFPV25-1 | *rpsM::gfp* mut | [6] |
| pKD3 | ApR, CmR, OriRγ | [7] |
| pKD4 | ApR, KmR, OriRγ | [7] |
| pKD46 | ApR, *oriR101*, *repA101*(ts), *araBp-gam-bet-exo* | [7] |
| pMPO20 | ApR, pWSK ∆ P*_Lac_* derived plasmid containing a modified MCS | This work |
| pMPO27 | ApR, expression vector with rrnBT1T2-Pm-*nasF* attenuator::MCS, ColE1 replication origin | [8] |
| pMPO51 | ApR, expression vector with rrnBT1T2-Pm-MCSII, ColE1 replication origin | This work |
| pMPO52 | ApR, expression vector with rrnBT1T2-Pm-T7 SD sequence-MCSII, ColE1 replication origin | This work |
| pMPO53 | ApR, expression vector with rrnBT1T2-Pm-T7 SD sequence-MCSII*-sspH2* signal peptide, ColE1 replication origin | This work |
| pMPO54 | ApR, expression vector with rrnBT1T2-Pm-MCSII, M13 replication origin | This work |
| pMPO55 | ApR, expression vector with rrnBT1T2-Pm-T7 SD sequence-MCSII, M13 replication origin | This work |
| pMPO56 | ApR, expression vector with rrnBT1T2-Pm-T7 SD sequence-MCSII*-sspH2* signal peptide, M13 replication origin | This work |
| pMPO57 | ApR, expression vector with rrnBT1T2-Pm-*nasF* attenuator-MCSII, ColE1 replication origin | This work |
| pMPO58 | ApR, expression vector with rrnBT1T2-Pm- *nasF* attenuator-T7 SD sequence-MCSII, ColE1 replication origin | This work |
| pMPO59 | ApR, expression vector with rrnBT1T2-Pm- *nasF* attenuator-T7 SD sequence-MCSII- *sspH2* signal peptide, ColE1 replication origin | This work |
| pMPO60 | ApR, expression vector with rrnBT1T2-Pm-*nasF* attenuator-MCSII, M13 replication origin | This work |
| pMPO61 | ApR, expression vector with rrnBT1T2-Pm- *nasF* attenuator-T7 SD sequence-MCSII, MCS M13 replication origin | This work |
| pMPO62 | ApR, expression vector with rrnBT1T2-Pm- *nasF* attenuator-T7 SD sequence-MCSII- *sspH2* signal peptide, M13 replication origin | This work |
| pMPO64 | *nahR/*P*_sal_–xylS–* CmR*-*P*_Tac_–gfp* | This work |
| pMPO83 | CmR*-*His t*-*P*_Tac_–gfp* | This work |
| pMPO94 | ApR, expression vector with rrnBT1T2-Pm-*nasF* attenuator-MCSII-*cat*::*lacZ*, ColE1 replication origin | This work |
| pMPO96 | ApR, expression vector with rrnBT1T2-Pm-MCSII-*cat*::*lacZ*, ColE1 replication origin | This work |
| pMPO200 | ApR. Broad host-range *lacZ* translational fusion vector, based on pBBR1MCS-4. | [9] |
| pMPO1000 | ApR, expression vector with rrnBT1T2-Pm-MCSII-*cat*::*lacZ*, M13 replication origin | This work |
| pMPO1001 | ApR, expression vector with rrnBT1T2-Pm-*nasF* attenuator-MCSII-*cat*::*lacZ*, M13 replication origin | This work |
| pMPO1003 | ApR, expression vector with rrnBT1T2-Pm-T7 SD sequence-MCSII-Ha epitope encoding sequence, ColE1 replication origin | This work |
| pMPO1004 | ApR, expression vector with rrnBT1T2-Pm-T7 SD sequence-MCSII*-sspH2* signal peptide-Ha epitope encoding sequence, ColE1 replication origin | This work |
| pMPO1005 | ApR, expression vector with rrnBT1T2-Pm-T7 SD sequence-MCSII-*lacZ*, ColE1 replication origin | This work |
| pMPO1006 | ApR, expression vector with rrnBT1T2-Pm-T7 SD sequence-MCSII-*lacZ*, M13 replication origin | This work |
| pMPO1007 | ApR, expression vector with rrnBT1T2-Pm- *nasF* attenuator-T7 SD sequence-MCSII-*lacZ*, ColE1 replication origin | This work |
| pMPO1008 | ApR, expression vector with rrnBT1T2-Pm- *nasF* attenuator-T7 SD sequence-MCSII-*lacZ*, MCS M13 replication origin | This work |
| pMPO1009 | ApR, pMPO27 derived plasmid with colE3 cloned downstream Pm | This work |
| pMPO1010 | ApR, pMPO20 derived plasmid with colE3 cloned downstream Pm | This work |
| pMPO1011 | ApR, pMPO20 derived plasmid with colE3 cloned downstream Pm and bearing *nasF* attenuator | This work |
| pMPO1035 | *nahR/*P*_sal_–xylS- nasR-*CmR | This work |
| pMPO1046 | ApR, expression vector with rrnBT1T2-Pm-T7 SD sequence-MCSII-dTomato, ColE1 replication origin | This work |
| pMP220 | TetR, promoter probe vector | [10] |
| pT7-7 | ApR, expression vector bearing the Ø10 gene promoter and ribosome binding site. | [11] |
| pUC18N*colE3* | ApR, pUC18*Not* cloning vector P*lac::colE3* | Provided by E. Diaz |
| pWSK29 | ApR cloning vector | [12] |

**REFERENCES.**

1. Hanahan D (1983) Studies on transformation of Escherichia coli with plasmids. J Mol Biol 166: 557-580.

2. Diaz E, Munthali M, de Lorenzo V, Timmis KN (1994) Universal barrier to lateral spread of specific genes among microorganisms. Mol Microbiol 13: 855-861.

3. Fields PI, Swanson RV, Haidaris CG, Heffron F (1986) Mutants of Salmonella typhimurium that cannot survive within the macrophage are avirulent. Proc Natl Acad Sci U S A 83: 5189-5193.

4. Shaner NC, Campbell RE, Steinbach PA, Giepmans BN, Palmer AE, et al. (2004) Improved monomeric red, orange and yellow fluorescent proteins derived from Discosoma sp. red fluorescent protein. Nat Biotechnol 22: 1567-1572.

5. Cherepanov PP, Wackernagel W (1995) Gene disruption in Escherichia coli: TcR and KmR cassettes with the option of Flp-catalyzed excision of the antibiotic-resistance determinant. Gene 158: 9-14.

6. Valdivia RH, Falkow S (1996) Bacterial genetics by flow cytometry: rapid isolation of Salmonella typhimurium acid-inducible promoters by differential fluorescence induction. Mol Microbiol 22: 367-378.

7. Datsenko KA, Wanner BL (2000) One-step inactivation of chromosomal genes in Escherichia coli K-12 using PCR products. Proc Natl Acad Sci U S A 97: 6640-6645.

8. Royo JL, Manyani H, Cebolla A, Santero E (2005) A new generation of vectors with increased induction ratios by overimposing a second regulatory level by attenuation. Nucleic Acids Res 33: e169.

9. Garcia-Gonzalez V, Govantes F, Porrua O, Santero E (2005) Regulation of the Pseudomonas sp. strain ADP cyanuric acid degradation operon. J Bacteriol 187: 155-167.

10. Spaink H, Okker R, Wijffelman C, Pees E, Lugtenberg B (1987) Promoters in the nodulation region of the Rhizobium leguminosarum Sym plasmid pRL1JI. Plant Molecular Biology 9: 27-39.

11. Tabor S, Richardson CC (1990) DNA sequence analysis with a modified bacteriophage T7 DNA polymerase. Effect of pyrophosphorolysis and metal ions. J Biol Chem 265: 8322-8328.

12. Wang RF, Kushner SR (1991) Construction of versatile low-copy-number vectors for cloning, sequencing and gene expression in Escherichia coli. Gene 100: 195-199.
