# Supplementary material for: Improved Expression Systems for Regulated Expression in Salmonella Infecting Eukaryotic Cells
Source: PLoS One. 2011 Aug 1;6(8):e23055. doi: 10.1371/journal.pone.0023055 (PMC3148252; doi:10.1371/journal.pone.0023055)
Supplement: Data S1 — Construction of expression vectors, lacZ fusions and colE3 cloning. (DOC) [file pone.0023055.s002.doc]

**Expression vector construction.**

KpnI and SacI sites were removed from the MCS of the high copy number pMPO27 plasmid by digesting with these enzymes, followed by blunt-end cloning back into the original vector. A 73 bp DNA fragment containing several restriction enzyme sites (MCSII) including an NdeI site that incorporates an ATG initiation codon, was digested with SmaI and HindIII and cloned into pMPO27 cut with the same enzymes, to generate pMPO57. A synthetic DNA fragment containing the T7 SD sequence was generated by annealing the overlapping complementary oligonucleotides SD pT7-7 +/-. This fragment was ligated to pMPO57 digested with SmaI and NdeI, to produce pMPO58. Genes cloned such that their initiation codon is inserted into the pMPO58 NdeI site maintain an optimal distance between the SD and the start codon.

The plasmids described above possess the *nasF* attenuator between the P*m* promoter and the cloning sites. To eliminate it, plasmids pMPO57 and pMPO58 were digested with EcoRI, which flanked the attenuator, and religated, thus generating plasmids pMPO51 (MCSII) and pMPO52 (MCSII-SD), respectively.

The pWSK29 vector was modified to construct a parallel collection of low-copy plasmids as follows. The P*_lac_* promoter was deleted by digesting with SacI and PshAI and religating the fragment after blunt-ending with T4 DNA polymerase. The NdeI site was eliminated by digesting with NdeI, filling-in with Klenow, and religated. The original multicloning site was also modified by cutting out the KpnI-ClaI fragment to remove some of the repeated site within the polylinker. The resulting plasmid was named pMPO20.

The expression modules with all their relevant features present in the plasmids pMPO51, pMPO52, pMPO57 and pMPO58 (rrnBT, Pm promoter and *nasF* attenuattor and/or T7 SD sequence when present), were cloned into pMP20 as a NotI-HindIII fragment. In this way low-copy plasmids pMPO54, pMPO55, pMPO60 and pMPO61 were constructed, keeping the same characteristics than their multi-copy counterparts (Figure 2).

We amplified the *sspH2* signal peptide coding region from *Salmonella enterica* serovar Typhimurium genomic DNA, using primers that contained NdeI and PstI restriction sites. The 450 bp amplified fragment was digested with these enzymes and cloned into the pMPO52, pMPO55, pMPO58 and pMPO61 expression vectors, to generate the pMPO53, pMPO56, pMPO59 and pMPO62 plasmid variants (See Figure 2).

pMPO1003 was constructed by cloning the synthetic SalI-HA-HindIII fragment into pMPO52.

**Construction of plasmids containing *lacZ* fusions.**

The *cat*::*lacZ* fusion from pMP220 was cloned into pMPO57 and pMPO51, generating the plasmids pMPO94 and pMPO96, respectively. In these vectors *lacZ* is translated from the SD sequence and the initiation codon from the *cat* gene. In addition, *lacZ* fusions with the translation initiation regions of plasmids pMPO58 and pMPO52 were constructed by joining the 8^th^ codon of *lacZ* from pMPO200 in frame with the ATG codon of the NdeI restriction site, generating the plasmids pMPO1007 and pMPO1005, respectively. Both types of fusions were transferred to the low-copy number plasmid pMPO20, as indicated above, to make the plasmids pMPO1000, pMPO1001, pMPO1008 and pMPO1006, respectively.

***colE3* cloning in expression vectors.**

A XbaI/blunted-HindIII 1.7 Kb DNA fragment carrying *colE3* was isolated from pUC18Not colE3 and cloned in pMPO54, pMPO57 and pMPO60 digested with SmaI-HindIII.
